# High-precision FRET analysis of the G-protein coupled receptor TGR5 in live cells

**DOI:** 10.1186/2047-783X-19-S1-S12

**Published:** 2014-06-19

**Authors:** Annemarie Koch, Qijun Ma, Manuel Frohnapfel, Lina Spomer, Verena Keitel-Anselmino, Christoph Gertzen, Holger Gohlke, Claus AM Seidel

**Affiliations:** 1 Institute for Molecular Physical Chemistry, Heinrich Heine University, 40225 Düsseldorf, Germany; 2 Clinic of Gastroenterology, Hepatology and Infectious Diseases, Heinrich Heine University, 40225 Düsseldorf, Germany; 3 Institute for Pharmaceutical Chemistry, Heinrich Heine University, 40225 Düsseldorf, Germany

## Background

TGR5 is a widely expressed and highly conserved G protein coupled receptor. Its activity and functionality is commonly modulated by bile acids, especially by lithocholic acid. As true for all ligand activated G protein coupled receptors a G protein subunit is released from TGR5 after ligand binding and initiates a signaling cascade resulting in a cell type specific response. Current investigations suggest an involvement of TGR5 in bile homeostasis, inflammatory responses and hepatobiliary diseases. Therefore a targeted therapy involving site specific inhibition of TGR5 is of immense interest. However, up to date no solved structure of TGR5 exists and oligomerization properties are largely unknown. To determine structural changes and oligomerization properties of TGR5 we designed a three way strategy including TGR5 plasmids coupled with (I) fluorescent proteins (FPs) at the C-terminus or (II) a N-terminal peptide tag (ACP) for subsequent labeling with a fluorescent dye and (III) unnatural amino acids (UAAs) for site specific extracellular labeling.

## Methods

Oligomerization properties were studied by high-precision Förster Resonance Energy Transfer (FRET) measured by multi-parameter fluorescence imaging spectroscopy (MFIS) using the labeling strategies (I) and (II) in live cells [1,2]. FRET is a mechanism describing energy transfer between two chromophores. A donor chromophore, initially in its electronic excited state, may transfer energy to an acceptor chromophore. The requirements are 1) overlap between donor’s fluorescence emission spectrum and acceptor’s absorption spectrum, and 2) transition dipole interaction between the two fluorophores. The efficiency of the energy transfer is inversely proportional to the sixth power of the distance between donor and acceptor making FRET extremely sensitive to small distances. Due to the fully recording of every single photon by the wavelength and the polarization, MFIS can simultaneously measure the fluorescence parameters including anisotropy, fluorescence lifetime and fluorescence intensities during live cell imaging over hours with picosecond resolution. During MFIS data processing FRET populations can be analyzed by plotting the signal intensity ratio between the green and red channels against the fluorescence lifetime [2]. Labeling strategy (I) was used to analyze intracellular interactions between TGR5 proteins fused to fluorescent proteins. The EGFP-mCherry pair shows good spectral overlap with a Förster radius of 51 Ångström and is therefore an ideal reporter pair. For labeling strategy (II) the N-terminal ACP peptide tag was labeled with either CoA-Atto488 or CoA-Cy3 as a donor or CoA-Cy5 as an acceptor. The Förster radii for Atto488-Cy5 and Cy3-Cy5 are 51 and 56 Ångström, respectively.

## Results

For strategy (I) cells were co-transfected with TGR5-GFP and TGR5-mCherry and analyzed with MFIS. Successfully transfected Hep2 cells clearly presented TGR5 coupled with fluorescent proteins at the cell membrane. An overlay of TGR5-GFP and TGR5-mCherry figures suggests co-localization of GFP and mCherry. Further analysis of these cells showed a reduced donor fluorescence lifetime of GFP compared to those only transfected with TGR5-GFP. This effect could be increased by acceptor titration of TGR5-mCherry (EGFP-mCherry from ratio 1:1 to 1:20). The shortened donor lifetime indicates that FRET occurs between TGR5-GFP and TGR5-mCherry probably due to TGR5 oligomerization. Consistent results were obtained with labeling method (II). For this purpose Hep2 cells were transfected with ACP-TGR5. The ACP-tag fused N-terminal to TGR5 was located extracellular and labeling was possible under live cell conditions by simply adding ACP synthase and CoA tagged dyes to the normal medium with further incubation for 1 hour at 37°C. The ACP-tag was labeled with CoA-Cy3 or CoA-Atto488 alone or in combination with CoA-Cy5. Live cell imaging suggests co-localization of ACP-tagged Cy3 and Cy5. The analysis of the extracellular labels clearly indicated a reduced donor fluorescence lifetime of Cy3 as compared to Hep2 cells labeled with CoA-Cy3 only or CoA-Atto488. This suggests intermolecular, extracellular FRET between ACP-TGR5 labeled with Cy3 and Cy5. To show, whether intramolecular FRET might occur, we used a combination of labeling strategy (I) and (II) and transfected cells with TGR5 N-terminally tagged with ACP and C-terminally fused to YFP. After 24h cells expressing ACP-TGR5-YFP were subsequently labeled with CoA-Cy3 or CoA-Cy5. However, intra-molecular FRET from the C-terminal YFP to the N-terminally labeled ACP tag could not be observed. As a control for oligomerization ACP-tagged ADRbeta2 was measured in parallel. CoA-Atto488 or CoA-Cy3 and Cy5-labeled ADRbeta2 co-localized at the cell membrane and energy transfer occurred from Cy3 to Cy5 and CoA-Atto488 to CoA-Cy5, respectively. We applied a quantitative analysis method on the MFIS-FRET data taking account of the double exponential decay characteristic of EGFP in live cells. The results showed that more than one FRET population exist in all the measurements, which suggest that TGR5 may form a higher-order complex than dimer. Also, the interacting pattern of TGR5 is distinctive comparing with 14 other receptor protein interactions we have previously measured and analyzed using the same method.

## Conclusion

With our labeling strategies (I) TGR5-FPs and (II) ACP-tagged TGR5 we showed that FRET proved TGR5 oligomerization. However, the stoichiometry remains to be determined. The structural properties (especially of flexible loops) and structural changes induced by ligand binding will be probed using the third labeling strategy. Based on homology models several amino acid positions in the extracellular loops were selected for site specific labeling. From overlap extension mutagenesis derived TGR5 mutants carrying an Amber stop codon will be used for incorporation of unnatural amino acids and further site specific extracellular labeling with tetrazine-dyes via copper free click chemistry. Preliminary results with a control GFP39TAG mutant showed successful incorporation of UAAs in live cells as verified by green fluorescence of the completely translated protein, which will otherwise not be fluorescent. TGR5, like other GPCRs, may form higher-order complexes which further complicates the analysis. Our goal is to utilize the high-precision FRET screening method to filter out the possible complex conformations of TGR5 [3].
